# From listening to action: how social and behavioral evidence informed multisectoral cholera response in Zambia, 2023–2024

**DOI:** 10.3389/fpubh.2026.1895133

**Published:** 2026-10-09

**Authors:** Soha Karam, Nadia Butler, Tikulirekuti Banda, Hanna Woldemeskel, Oliver Mweemba, Mulanda Joseph Mulawa, Kalangwa Kalangwa, Leslie Jones, Anastasiia Atif, Elisabeth Ganter Restrepo, Yves Ngaleu, Maureen McKenna, Rachel James, Juliet Bedford, Helen Smith

**Affiliations:** 1 Anthrologica Ltd, Oxford, United Kingdom; 2 UNICEF Country Office, Lusaka, Zambia; 3 Department of Health Promotion and Education, The University of Zambia, Lusaka, Zambia; 4 Ministry of Health, Lusaka, Zambia; 5 United Nations Children’s Fund (UNICEF) Global Programme Division, Nairobi, Kenya; 6 International Federation of Red Cross and Red Crescent Societies (IFRC), Africa Regional Office, Nairobi, Kenya; 7 International Federation of Red Cross and Red Crescent Societies (IFRC), Country Cluster Delegation for Zimbabwe, Zambia and Malawi, Harare, Zimbabwe; 8 (Formerly of) Risk Communication Community Engagement (RCCE) Collective Service, Geneva, Switzerland

**Keywords:** cholera, community feedback mechanism, outbreak response, rapid qualitative assessment, risk communication community engagement, social and behavioral science

## Abstract

Rapid qualitative assessments and community feedback mechanisms are increasingly recognized as core components of risk communication and community engagement in public health emergencies, yet few documented examples exist of how this evidence has directly influenced outbreak response decision-making. This case study examines the 2023–2024 cholera outbreak in Zambia, analyzing how social and behavioral data generated through multiple rounds of rapid qualitative assessment, alongside national community feedback mechanisms, informed multisectoral response activities. Drawing on document review, consultations with response pillar members, assessment reports, feedback dashboards, and triangulation with epidemiological data, the study traces how community concerns, rumors, beliefs, perceptions and priorities were collected, analyzed and applied across health, water, sanitation and hygiene, and protection response pillars. Enabling factors included pre-existing technical capacity, strong partnerships between government humanitarian actors, and academia, and rapid feedback loops that supported near real-time adaptation. Challenges at different points in the response included delayed resource allocation, uneven subnational coordination, and limited capacity to process large volumes of feedback data. The Zambia experience demonstrates that community-generated evidence can meaningfully influence outbreak response when integrated across response pillars rather than confined to behavioral interventions alone, representing a broader shift in public health practice that positions community perspectives as central to decision-making. This, in turn, strengthens effectiveness, trust, accountability, and transparency by aligning interventions with community-identified priorities. While grounded in the Zambian context, the findings are relevant for preparedness, anticipatory action, and response across a range of public health emergencies.

## 1 Introduction

Despite growing recognition of the importance of Risk Communication and Community Engagement (RCCE) in global guidance and the international health regulations for public health emergencies (1), RCCE is often operationalized as a downstream communication function rather than as an essential component of public health emergency response decision-making. In emergency contexts, the collection and analysis of social and behavioral data by RCCE practitioners through engaging communities is still perceived as prohibitively expensive, methodologically complex, and too time-consuming to inform time-sensitive interventions.

This tension became most apparent during the COVID-19 pandemic. Global guidance consistently emphasized the role of RCCE in facilitating timely information provision, addressing misinformation, and increasing public acceptance of and adherence to public health and social measures (2). International agencies, including UNICEF and the European Center for Disease Prevention and Control (ECDC), published practical technical guidance to support the operationalization of RCCE at the country level (3). At the same time, the Collective Service [a partnership, focusing on RCCE, between the International Federation of Red Cross and Red Crescent Societies (IFRC), UNICEF, WHO, and the Global Outbreak Alert and Response Network (GOARN)] released an updated global strategy for RCCE that began to shift the dominant narrative from one-way directive communication toward participatory community engagement approaches aimed at strengthening response effectiveness (4).

More recently, WHO guidance on strengthening health emergency prevention, preparedness, and response in health emergencies has explicitly positioned RCCE as an essential component of community protection to empower communities and strengthen resilience during health emergencies. However, emerging evidence suggests that RCCE is rarely implemented in this way in practice (5). In particular, during disease outbreaks, the urgency of response activities is often assumed to preclude the generation and use of rapidly generated qualitative data and community feedback capable of informing intervention design and implementation. As a result, this research is sometimes treated as optional—valuable but non-essential—rather than as a core response function. To strengthen outbreak decision-making, social and behavioral evidence should be understood as a core public health intelligence stream that generates real-time operational insights, complementing epidemiological, surveillance, and water, sanitation and hygiene (WASH) data, to guide rapid adaptation across all technical pillars.

A key objective of the Collective Service RCCE strategy is to promote data-driven RCCE. For this to work, decision-makers need to plan for the systematic collection and analysis of data on community perceptions, behaviors, concerns, and social dynamics, triangulate it with other data sources, and establish processes to translate insights into operational actions with and for relevant response actors. However, in the fast-paced reality of public health emergencies, setting up and sustaining such evidence-driven processes for RCCE remains challenging (6). At the same time, the capacity to generate social and behavioral insights during emergencies is often limited, and even when data are available, analysis and use are frequently not prioritized.

In practice, evidence for RCCE is generated through a range of research methods, including population-based knowledge, attitude and practice surveys; online and offline social listening and community feedback mechanisms (CFMs); community mapping and situation analysis; and rapid assessments, including rapid qualitative assessments (RQAs). The use of these methods, particularly rapid and qualitative approaches, has expanded recently, accelerated by the COVID-19 response, which reinforced the operational value of timely community-generated insights. As a result, there is now extensive operational guidance to support the collection of this data across a range of infectious disease outbreaks and public health emergencies (7–9). However, implementation experience suggests that less attention has been paid to how this data is routinely analyzed, integrated, and systematically used to inform decision-making across response pillars.^1^

This case study examines how data-driven RCCE contributed to broader outbreak response outcomes during the 2023–24 cholera outbreak in Zambia. The case study draws on findings from a retrospective study commissioned by the Collective Service, which analyzed data generated through RQAs and U-report polls, and community feedback items collected through mechanisms such as hotlines and call centers, to show how community-generated insights were used in real-time to adapt RCCE strategies to the Zambia context. A contribution analysis, part of the retrospective study conducted after the outbreak was under control, assessed the role of these adaptations in shaping response processes and outcomes. The retrospective study did not set out to establish direct attribution, but to develop a credible, evidence-informed narrative of contribution, test the plausibility that RCCE evidence informed response activities, and consider alternative influences that may have shaped outbreak outcomes.

This case study focuses on how the acceptance and integration of evidence-based RCCE fostered a culture of learning and responsiveness across the response pillars, strengthening the overall effectiveness of the cholera response.

## 2 Context of the cholera response

Cholera has been endemic in Zambia since 1977 (10). The incidence of cholera is driven by multiple factors, among the most critical of which are water and sanitation (11). Over 6 million Zambians lack access to safe drinking water, and 11 million lack access to basic sanitation facilities (12). The country’s health system is under-resourced. There are significant disparities in healthcare access between urban and rural areas, and the country lacks capacity in terms of disease surveillance, laboratory capacity, and resources for outbreak detection and response (13). High population density coupled with limited socioeconomic activities in unplanned settlements further exacerbates the situation.

The 2023–24 cholera outbreak in Zambia was the largest recorded. The outbreak began in October 2023 in the peri-urban areas of Zambia’s Lusaka province and spread to Central, Northern, Copperbelt, North-Western, Southern, and Eastern provinces due to high transmission rates and cross-border trade (14). By the end of January 2024, all 10 provinces had reported cases of cholera. Lusaka remained the epicenter, accounting for over 90% of all reported cases in the country at the peak of the outbreak (15).

By June 2024, over 23,000 cases and 740 deaths had been reported. The case fatality rate (CFR) was 3.17% as of June 2024; while this was lower than the CFR during the early phase of the outbreak (October 2023-mid-January 2024), it still exceeded WHO’s expected threshold of 1% or lower (16). Most deaths occurred outside health facilities, indicating that affected people were not seeking health interventions (17). Concurrently with the cholera outbreak, the country experienced an anthrax outbreak and severe drought, placing additional pressure on already-strained health systems and communities.

The Zambia National Public Health Institute (ZNPHI) under the leadership of the Disaster Management and Mitigation Unit (DMMU) in collaboration with the Ministry of Health (MoH) led the cholera response, supported by national and subnational response Incident Management System (IMS) coordination structures. The IMS coordination structure comprised 10 pillars: Leadership and Coordination; Surveillance; RCCE; Points of Entry; National Laboratory System; IPC and WASH; Case Management and Health System Strengthening; Maintaining Essential Health Services; Operations, Supply Chain and Logistics; and Vaccinations (18).

Recognizing RCCE as central to effective outbreak response, the national RCCE Pillar was activated in October 2023. Co-chaired by the MoH Health Promotions and Community Health Unit and the ZNPHI with technical support from UNICEF Zambia Country Office, the pillar comprised five sub-pillars: (i) coordination and communication; (ii) risk communication systems; (iii) public communication; (iv) dynamic listening and research; and (v) community engagement. Communication and engagement ensured harmonized planning and information sharing across stakeholders and line ministries. Risk communication strengthened preparedness, protocols, and integration of risk communication at national and subnational levels. Public communication oversaw key multisectoral messaging and media engagement. The dynamic listening and research sub-pillar generated real-time community insights through community feedback mechanisms and rapid qualitative assessments to inform messaging, engagement activities, and multisectoral response planning and was chaired by MoH with technical support from UNICEF. Community engagement coordinated subnational activities and engagement of at-risk groups.

The cholera emergency both tested and strengthened this capacity, particularly the dynamic listening and research pillar, which became central to generating community insights that informed not only the RCCE pillar but the broader 2023–24 multisectoral cholera response.

## 3 Key programmatic details

### 3.1 RCCE activities as operational research in practice

The main functions of the RCCE response pillar included engaging communities to increase preventive behaviors and promoting early care-seeking; promoting the use of oral rehydration salts (ORS) at community and household level; conducting assessments of knowledge, attitudes, and practices on cholera; strengthening health promotion efforts and local structures for RCCE; and building capacity for health workers, community volunteers, community leaders, influencers, and media on cholera control and risk communication.

From October 2023 onwards, RCCE efforts intensified, including disseminating existing Information, Education and Communication (IEC) materials, mapping priority communication channels, and creating a multimedia distribution plan for RCCE messaging. During the first few months of the outbreak, the focus was on addressing knowledge gaps on cholera transmission, prevention and the need for early treatment. RCCE interventions during this period included communication of information about cholera to the public via multiple channels already in use, including public address systems, phone, radio programs, television programs, social media (Instagram and Facebook), drama performances in markets, distribution of stickers and posters, and community engagement activities (e.g., deployment of community-based volunteers (CBVs) to households) (18–22).

As the outbreak intensified, greater emphasis was placed on systematically listening to communities and integrating feedback into response processes. Despite sustained cholera prevention messaging through multimedia campaigns and community-based volunteers, transmission and community deaths remained high, driven by delayed care-seeking. While the RCCE pillar faced pressure to increase messaging on early treatment, there was a need to understand why people were not presenting earlier.

To address this, the dynamic listening and research sub-pillar conducted four rounds of RQAs in cholera hotspot communities. Led by UNICEF Zambia, with technical surge support from UNICEF Eastern and Southern Africa Regional Office (ESARO) and the Collective Service, the assessments included almost 200 key informant interviews with community members, cholera patients and survivors, health workers, volunteers, and traditional and religious leaders, adolescents, men and women, and Kachasu (local home brew) brewers. Early findings highlighted complex social, structural and trust-related barriers extending beyond gaps in knowledge of cholera prevention and treatment. These insights informed adaptations and refinements to risk communication and messaging; dissemination continued through the same broad range of channels, with intensified reach over the course of the response.

In parallel, an interactive cholera community feedback dashboard was established by the Collective Service with technical support from IFRC and operationalized by UNICEF Zambia through partners including the Zambia Red Cross Society (ZRCS), Zambia Interfaith Networking Group (ZINGO), Support to Older People (STOP) and Lifeline/Childline. Between January and December 2024, the dashboard captured 2,843 items of community feedback from all 10 provinces, primarily community requests for chlorine (76%), waste disposal (5%), vaccines (4%), ORS (3%) and water tanks (2%), alongside questions about cholera symptoms, treatment and vaccine effectiveness. The dashboard supported the RCCE pillar and provided relevant insights for other response pillars by aggregating social and behavioral data from multiple community feedback mechanisms (23, 24).

A second dashboard, developed by the ZRCS with IFRC support, operated throughout the response from February 2024. Together, these community feedback mechanisms collected and categorized over 17,000 items, including requests for essential supplies and services, as well as community perceptions, beliefs and concerns. These data provided actionable social and behavioral insights that were used to refine and adapt response activities over the course of the outbreak.

### 3.2 Contribution analysis

After the outbreak was brought under control, a retrospective qualitative study of the cholera response was commissioned by the Collective Service (25), to explore the possible link between RCCE activities and response and health outcomes. The study also included a temporal analysis of epidemiological data trends before, during and after RCCE activities to explore the contribution of RCCE to health outcomes; while not included in this case study, the figure showing the alignment of epidemiological trends with the timing of RCCE activities across the entire outbreak timeline is provided in Supplementary file 1.

The study relied on secondary data generated between October 2023 and September 2024 (RQA data, CFM items, interview and meeting notes, donor and internal reports, epidemiological data, and contextual and other documentation), and consultations with key RCCE actors involved in the cholera response. Most of this data was contained within unpublished, internal sources generated rapidly during the outbreak response and shared freely by the organizations and individuals who collaborated on and co-authored the retrospective study. Publicly available data (e.g., published situation analysis documents and strategy documents) are cited in the reference list; unpublished internal sources (e.g., RQA reports, interviews, CFM dashboard data, epidemiological datasets) are identified as such in the reference list. For the retrospective study, we thematically analyzed the data from the four rounds of RQA, the CFM dashboards (including time-stamped, geotagged records of issues raised, response actions and categories of concern), and interviews conducted with key response partners during the response. We also conducted consultations with national and subnational stakeholders helped to identify examples of how RQA findings and community feedback were used during the cholera response. Initial findings from the retrospective study were validated with UNICEF, government, and other response actors. This level of careful triangulation across multiple sources served as the primary validation of themes and findings in the data.

This community case study builds on findings from the retrospective study to highlight how community-generated insights were used in real-time to adapt RCCE strategies to the Zambia cholera outbreak. The next section draws on the thematic and contribution analysis conducted for the retrospective study to highlight examples of how community insights were used to directly inform response decision-making. The implementation learning section reflects on the various analyses conducted in the retrospective study to identify the factors that enabled and constrained use of community insights during the response. Finally, the implications section of this case study draws directly on the implications and recommendations of the retrospective study, which were refined at the time in a validation meeting with Collective Service partners, UNICEF, and key national stakeholders.

## 4 From listening to action: how community insights led to action across response pillars

This section examines how social and behavioral evidence generated during the cholera response was translated into concrete actions across the pillars. Drawing on the thematic and contribution analysis within the wider retrospective study, we identify the main pathways through which community insights informed decision-making and adaptive response processes. We first summarize the main categories of evidence use, before presenting a more detailed example illustrating how community-generated insights informed RCCE activities and contributed to improved behavioral outcomes.

The contribution analysis identified 39 distinct examples where community insights from RQAs and CFM items were used to inform response decisions. These examples are based on operational actions taken, documented in multiple data sources (see Supplementary file 2). These examples, were grouped into four broad categories: (a) informing risk communication and messaging; (b) informing the development or adaptation of RCCE activities and capacities; (c) informing multisectoral programming and operational decisions; and (d) informing policy decisions. Table 1 provides selected examples of how community insights, derived from RQAs and community feedback mechanisms, were translated into response actions.

**Table 1:** Examples of the use of community insights in response actions.

Data source/feedback type	Insight	Action taken
Informed risk communication and messaging		
RQAs	Communities had some correct knowledge about cholera and had heard a range of cholera prevention guidance, but often the greatest emphasis was on a clean environment and there were knowledge gaps on the connection between unsafe water, early care seeking behaviors and cholera prevention	The “3Cs” information campaign was jointly developed by UNICEF, MoH and ZNPHI, focusing on clean water, clean hands and early care-seeking. A poster was printed and distributed widely in Lusaka and other provinces. The shift to focusing on 3Cs has been emphasized in MoH statements about the outbreak
Door-to-door visits by CBVs, CHWs and doctors	People were confused about when to present to the health facility because messaging was focused on the last symptom rather than the first	The health message was changed to focus on the first symptom of cholera, in a culturally acceptable way
RQAs	Community reluctant to seek care at Cholera Treatment Centers (CTCs) due to fears of dying. People’s reasons for fearing CTCs varied by location, but were in some cases fueled by fear campaigns on social media	Targeted education and messaging campaigns were developed across pillars, including the case management pillar, to build trust in CTCs and encourage early care-seeking. CBV job aids were developed with information on what happens at CTCs and Oral Rehydration Points (ORPs)
RQAs	There were misconceptions within the community about cholera transmission and the use of traditional remedies/prevention methods such as kachasu (local home brew)	The RCCE pillar tailored health communication messages and created IEC materials, including clarifying transmission routes and promoting prevention and treatment methods like ORS. CBVs’ Q&A tool and training materials were updated and child-friendly posters about the use of kachasu distributed in schools
Informed adaptation of RCCE activities		
Door-to-door visits by CBVs	Although a high-risk group, it was noted that men were not involved in CBV door-to-door visits as they are not home during the day	The RCCE response plan and budget were adjusted to include strategies to reach men with risk information. Subsequent rounds of RQAs were conducted with men in affected communities, and the insights were used to inform programming, including targeted interventions for men conducted in hotspots together with the DL&R pillar. ZRCS and partners conducted sensitization and distributed IEC materials in public spaces
RQAs		
RQAs	Specific needs, challenges and barriers existed for certain groups, such as fishermen	Community dialog meetings were held with at-risk communities to understand trusted channels of communication as well as preferred times for engagement
RQAs	IEC materials were inaccessible for people living with disabilities (PLWDs) (e.g., deaf and blind)	A co-creation workshop was held with disability networks and organizations of PLWDs, including the Zambia Deaf Society and STOP, resulting in RCCE materials: OCV poster, ORP and OCV job aids, cholera brochures, and translated 3Cs posters (braille and sign language), all distributed in hotspot areas. Revised multimedia messages were disseminated via 15 national and community radio/television stations with over 10 million estimated listeners. STOP introduced a sign language interpreter for TV dissemination
Informed multisectoral programming and operational decisions		
RQAs	Community members highlighted the transportation barriers they faced in reaching CTCs, including lack of transportation, impassable roads and safety concerns. Initially the only CTC was at Heroes’ Stadium in Lusaka, and people were afraid to go there	A transport funding scheme was developed and transportation provided to help facilitate access to healthcare. The MoH and UNICEF paid to rehabilitate 7 ambulances in Lusaka for patient transport and provided fuel. Other partners purchased additional vehicles, working with private car hires. Transport was also provided to patients upon discharge. MoH also created more CTCs and ORPs to make services more accessible for people in communities and to reduce fear in accessing these. CBVs were able to refer suspected cases to facilities
RQAs	Although there was high acceptance of the OCV in principle, most people were unaware of the vaccine campaign and were therefore often missed during OCV delivery, particularly men	Men and young people were engaged with OCV sensitization via targeted activities (e.g., in bars, bus stations and schools) and older people were engaged through STOP Zambia
RQAs	People were unable to purchase soap and chlorine	UNICEF immediately procured chlorine and soap as part of Stop Cholera kits and provided the chlorine and soap to Red Cross Volunteers for distribution. The WASH team distributed chlorine packages. Other partners such as ADRA also distributed hygiene kits, including chlorine bottles
Informed policy decisions		
CFM	Communities were using pit latrines and shallow wells, which were contributing to the spread of cholera	A policy directive was issued to mandate the emptying of pit latrines and burying shallow wells in cholera hotspot areas, supported by civil society contributions of chlorine and fuel to expedite the exercise, while the government continued to provide water tanks as an alternative safe water source
RQAs	People were dissatisfied with being excluded from burial rituals, with many families feeling they could not grieve appropriately. The MoH faced challenges in identifying deceased bodies	According to the Interagency RCCE Action Tracker, the national safe and dignified burial guidelines were revised by the MoH to allow a select number of family members to participate in burial rituals and this change was communicated to communities. We do not have written evidence of this policy change. This change also aimed at enabling families to help identify deceased bodies, as identification had been challenging without their presence. The establishment of an information/help desk at Heroes Stadium also assisted with identification
CFM	Community members in congregate settings (e.g., markets, churches and schools) were not consistently adhering to public health measures to prevent the spread of cholera, and the MoH faced challenges containing transmission in these settings	A policy revision of the Public Health Act was made to require the provision of hygiene promotion buckets and soap in congregate settings (e.g., churches, markets and schools)

RQAs and CFM data prompted new or refocused messages, updated IEC materials (including versions for people with disabilities, children, older people, refugees, and different language groups), and government dissemination through ministerial statements and multiple channels (radio, TV, social media, household visits). For example, when RQAs found communities using kachasu (home brew) to treat cholera, the RCCE pillar developed materials promoting ORS instead; and when evidence from the CFM dashboard showed communities did not know how to use chlorine correctly, new instructional posters and public service announcements were produced.

Evidence also strengthened RCCE activities and capacities, for example feedback showed that men were missing RCCE engagement (because they were often away when volunteers visited), which led the RCCE pillar (together with the dynamic listening and research sub-pillar) to target outreach in bars, markets, and churches. Similarly, evidence of geographic RCCE capacity gaps led to focused RCCE training in border districts and cholera hotspots, such as Luapula province.

Multi-sectoral programming and operational decisions were also informed by community insights, often through cross-pillar or government uptake. This included improving access to health services (transport to CTCs, expanded OCV distribution hours and locations), addressing socio-economic barriers (cash transfers, hygiene kits), improving WASH (chlorine and soap distribution, burying shallow wells, water bowsers, toilet construction), strengthening psychosocial support, and reinforcing feedback mechanisms such as help desks at CTCs. For instance, evidence of cost-related barriers to care from RQA’s led UNICEF and partners to fund transport and activate cash transfers, while other RQA findings that showed that men wanted but could not access OCV led to expanded door-to-door and after-hours distribution.

There were two identifiable policy changes linked to insights from CFM data and RQA findings, though most reported policy influence lacked documentation. The MoH revised national burial guidelines to allow limited family participation after RQAs revealed distress over exclusion from burial rituals, and a policy directive mandated burying shallow wells and emptying pit latrines after feedback linked these practices to cholera transmission.

### 4.1 Adaptation pathways in action: the 3Cs to stop cholera campaign

This section presents a more detailed illustration of the evidence-to-action pathway—where community insights informed the adaptation of RCCE activities during the cholera outbreak in Zambia. It traces the pathway from data collected in RQAs and information derived from community feedback mechanisms to concrete, multisectoral actions, using the “3Cs to Stop Cholera” campaign as an example of how RCCE functioned as an enabling determinant of response effectiveness.

#### 4.1.1 The insight

Prior to the first round of RQAs, cholera-related messaging was broad in scope, with a strong emphasis on environmental cleanliness and sanitation (17, 26–29). Early RQAs (16–20 January) and community feedback showed that, although communities were generally aware of cholera, many struggled to act on the multiple prevention messages being disseminated. Messaging had focused on environmental cleanliness, yet community members often did not perceive its relevance to their circumstances (28, 30–32). RQAs also identified misconceptions about transmission and treatment. Unclean surroundings were commonly cited as the main cause of cholera, while safe drinking water and handwashing were mentioned less consistently (13, 33, 34). Delayed care-seeking was linked to transport barriers and fear of dying at the CTC without family notification. Some individuals reported relying on home-based remedies, including kachasu, believed to prevent or cure cholera (34).

#### 4.1.2 The decision

The RQA findings prompted a shift from broad sanitation messaging to a prioritized focus on clear, actionable behaviors. This informed the “3Cs to Stop Cholera—Clean and Safe Water, Clean Hands and Early Care.” Campaign (35), promoting water treatment, handwashing with soap, and prompt care-seeking with ORS use. The campaign’s rationale was that, in emergency contexts, simple, clear and prioritized messages are more likely to be understood and acted upon (17, 26, 27, 36, 37). Supporting measures included revised CBV job aids and IEC materials and combining community engagement activities with distribution of chlorine and soap to support behavior change.

#### 4.1.3 Adapting the response

The “3Cs to Stop Cholera” campaign messages were developed quickly in mid-January 2024, with the development of the broader campaign materials from January–April 2024 (17, 26, 27, 36, 38). Core materials included a Zambia-specific poster (see Figures 1, 2) and accompanying radio and television content. Child-friendly materials were jointly produced by the RCCE, WASH, and education sectors (33). To enhance inclusivity, disability organizations and key stakeholders contributed to development. Materials were translated into seven local languages and adapted into braille and sign language formats (24, 26, 27, 39, 40).

**Figure 1:**
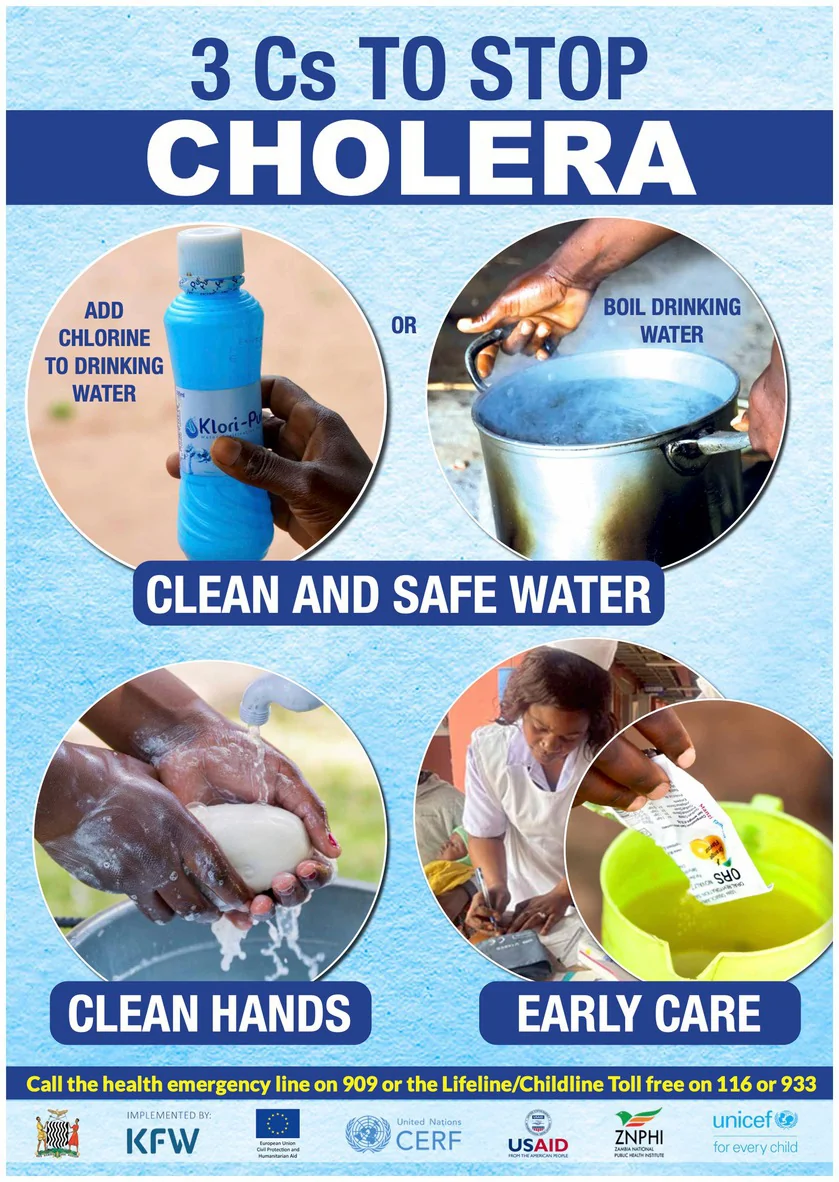
3Cs to stop cholera poster developed by the RCCE pillar. Image courtesy of UNICEF Zambia. Copyright © UNICEF Zambia.

**Figure 2:**
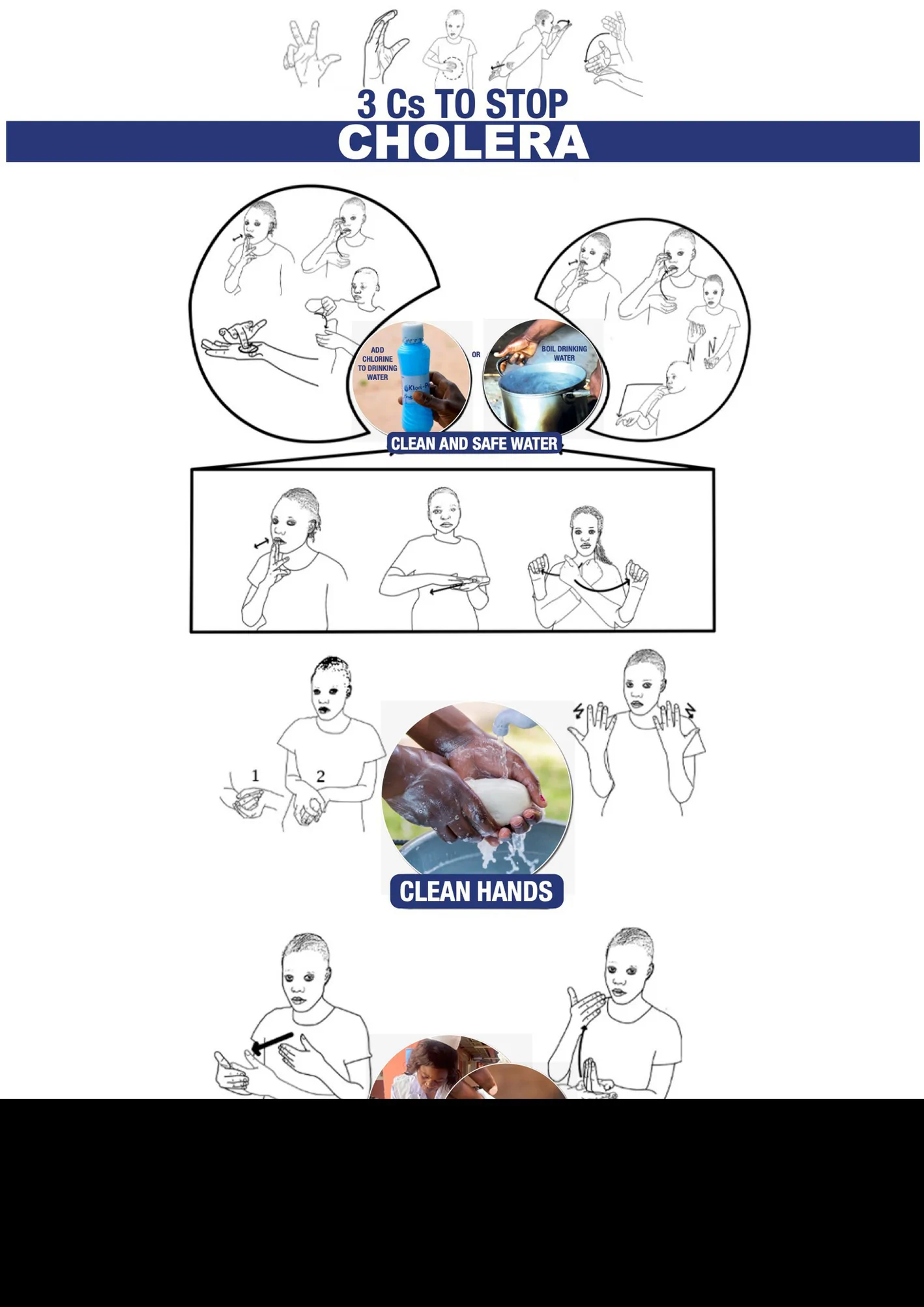
Disability friendly version of the 3Cs to stop cholera poster developed by the RCCE pillar. Image courtesy of UNICEF Zambia. Copyright © UNICEF Zambia.

#### 4.1.4 From messaging to multisectoral action

The 3Cs materials were rapidly printed and widely distributed in health facilities, CTCs, and public places across Zambia. Broadcast through 15 national television channels and community radio stations, the campaign achieved wide reach (26, 27). A complementary social media campaign launched on Instagram and Facebook reached approximately 243,000 people (41). The MoH integrated the 3Cs messages into daily briefings and press releases, reinforcing consistent public communication (17, 18, 26, 27, 42, 43). The community insights also informed operational decisions across pillars: teachers were trained on the 3Cs and supplied with WASH materials (33); chlorine and soap were distributed to households; transport funding was introduced to improve access to CTCs; and tailored messaging addressed fear and mistrust of facility-based care (35). These coordinated multisectoral actions aligned communication with practical support, enabling communities to adopt promoted behaviors (36).

#### 4.1.5 Contribution to response outcomes

When RCCE teams returned to communities following dissemination of the 3Cs campaign, partners reported improved understanding of cholera prevention and treatment messages and early indications of behavior change. Anecdotal evidence suggested adoption of preventive behaviors such as use of safe water, handwashing with soap, and earlier care-seeking to protect family members (13, 34, 36, 37, 44).

While changes in behavior cannot be attributed to RCCE interventions alone, the evidence generated through community feedback and RQAs, and the resulting 3Cs campaign, is likely to have contributed to improved understanding, prioritization and uptake of key behaviors (32, 44). This example illustrates how timely, community-generated insights enabled multisectoral action and strengthened the overall effectiveness of the outbreak response.

## 5 Implementation learning: what made community insights actionable?

Reflecting on the various analyses conducted in the retrospective study, this section summarizes key implementation learning from the Zambia cholera response. It highlights the factors that enabled community insights to be actionable, as well as roadblocks, constraints and challenges. This learning offers insights relevant to future emergency response planning beyond the specific context of cholera in Zambia.

### 5.1 Enablers

#### 5.1.1 Recognizing the value of social and behavioral evidence, especially that generated through RQAs and CFM

At the outset of the response, decision-making relied primarily on quantitative data, and the operational relevance of qualitative social and behavioral evidence was largely unrecognized. Over the course of the cholera response, the value of RQAs became evident, and they were adopted as a first-line analytical tool. RQAs subsequently shaped RCCE priorities and resourcing for later emergencies in Zambia, including the drought response and the 2025 Mpox outbreak.

#### 5.1.2 Rapid feedback loops, enabled through CFM dashboards and hotline data, supported real-time adaptation of RCCE activities

These mechanisms allowed response teams to adjust messaging and engagement strategies in response to emerging community concerns, including messaging on chlorine use, ORP locations, and other priority behaviors. The experience suggests that maintaining and routinely using these mechanisms, both during and outside outbreaks, is critical to sustaining RCCE practice. Engagement of affected communities and generation of community-level evidence are increasingly being integrated in response approaches, with qualitative social and behavioral evidence recognized as a standard component of humanitarian response in Zambia, as demonstrated during the drought, Mpox and measles responses.

#### 5.1.3 Impromptu technical collaborations between government actors and experts

Technical experts deployed from multiple agencies during the 2023–2024 cholera outbreak were key to legitimizing social and behavioral evidence and facilitating its real-time uptake. WHO and UNICEF ESARO staff were deployed to UNICEF Zambia, MoH, ZNPHI, and ZRCS, and experts from the UK Health Security Agency (UKHSA), US Centers for Disease Control and Prevention (CDC) and Collective Service also deployed to support the response. The in-country technical team proactively leveraged opportunities for dialog with government counterparts and response partners, to share emerging evidence and build trust-based working relationships. Several key response decisions and actions stemmed from these exchanges, contributing to increased visibility and credibility of RCCE within the response architecture. While not always predictable or easily replicated across outbreaks, this experience highlights the importance of informal collaborations alongside formal coordination structures and frameworks for enabling the use of evidence in policy and practice.

#### 5.1.4 Strong pre-positioned partnerships improved evidence generation and use

A pre-positioned network of technical and community-based actors was a key enabler of evidence generation and use. National institutions such as the University of Zambia (UNZA) provided expertise to support the rapid collection, analysis and synthesis of data to inform response decision-making. This capacity was complemented by a well-established network of community-based volunteers mobilized through ZRCS, extensive community reach through international NGOs (e.g., ZINGO), and the MoH’s health promotion and communication workforce, including Health Promotion Officers at national, provincial, district, and facility levels. Together, these actors enabled RCCE-informed decisions to be translated into operational actions.

### 5.2 Roadblocks and constraints

#### 5.2.1 Fragmented RCCE coordination and funding across government ministries

During the cholera response, RCCE responsibilities were distributed across multiple ministries with complementary mandates. The Ministry of Health coordinated the RCCE pillar within the public health response, support for mass media messaging was channeled through the Ministry of Information and Media, and community engagement activities through the Ministry of Community Development and Social Services. This created coordination challenges, as the lead technical coordinating ministry (Ministry of Health) did not receive dedicated resources to implement RCCE activities directly. A similar situation was observed during the drought response, highlighting a recurring structural issue.

#### 5.2.2 Inconsistent engagement of vulnerable and minority groups across the RCCE response

Some efforts began relatively early, particularly with networks of PLWDs through direct engagement, consultation, and co-creation of messages. However, accessible RCCE materials were finalized and distributed later, and engagement was not equally early, continuous, or consistent across all vulnerable and minority groups, including refugees. This limited the timely reach and effectiveness of adapted RCCE materials and activities for some populations.

#### 5.2.3 Inconsistent coordination and unclear leadership

While coordination structures and terms of reference for the RCCE pillar existed at national level, coordination was not systematic, particularly at subnational level. Although coordination improved during the response, it remained challenging at times. While the RCCE pillar was activated in October 2023 and a draft RCCE strategy was in place (later revised into a multi-hazard RCCE strategy), coordination mechanisms were not always operationalized, particularly at the subnational level. The RCCE subgroups were slow to be activated at subnational level, delaying the identification of priority actions and mobilization of partners in high-risk geographic areas. Roles, responsibilities, and strategic priorities were not always clearly communicated, which constrained timely sharing of community-generated evidence with response decision-makers, led to duplication of effort among partners, and created uncertainty over accountability for driving specific activities and follow-up. The coexistence of the Incident Management System public health RCCE subcommittee (led by the MoH/ZNPHI) and the newly established RCCE cluster under the drought response created parallel structures that were insufficiently linked, resulting in missed opportunities for coherence across plans and activities. Coordination was further weakened as cholera cases declined, and attention shifted to the drought response.

#### 5.2.4 Gaps between messaging and practical support

The initial response placed emphasis on sensitization and knowledge-based messaging, while the provision of practical enabling support such as provision of soap, chlorine, and infrastructural improvements was delayed. This created a disconnect between messaging and lived realities, with communities unable to act on the recommended behaviors despite increased awareness.

##### Greater focus on risk communication than community engagement

Risk communication activities such as megaphones and door-to-door information provision were prioritized by many response actors and, while they remain key components of RCCE, they were not consistently informed by community data from RQAs and the CFM. This reflects a critical gap in using community-generated evidence to shape more effective interventions.

##### Weak integration of RCCE with WASH and health, especially in coordination, research, and response

Although evidence gathered through RQAs and community feedback provided critical insights on care-seeking delays, ORS use, fear of CTCs, and water access, this evidence was not always consistently or rapidly integrated into health and WASH technical planning, particularly early in the response. While there were examples of community feedback and evidence being integrated quickly (e.g., adaptation of chlorine messaging and distribution, establishment of additional ORPs, and transport support for referral), parallel planning processes and siloed coordination structures meant that some insights (e.g., need for additional ORPs or improved chlorine distribution) were implemented later than was optimal.

#### 5.2.5 Inadequate resourcing for RCCE health-focused activities

Adequate resources were not allocated rapidly enough to RCCE at the start of the outbreak, particularly through government channels, leading to delays in some key activities. While partner support was mobilized relatively quickly, RCCE implementation still faced resource constraints that affected the scale and continuity of some community-level activities. These included the training and remuneration of CBV, as well as procurement of materials, and logistics to support RCCE implementation. This reflects a broader challenge in emergency response, where RCCE is often under-resourced relative to other response functions.

#### 5.2.6 Limited capacity for collecting, analyzing, using, and tracking CFM data

Community feedback data was not consistently collected or systematically entered, coded, and analyzed within the interagency dashboard. Gaps in information technology infrastructure and connectivity further constrained timely use of this feedback. These challenges highlight the need to formalize feedback mechanisms, at community, subnational, and national levels, and sustained investment to ensure feedback is routinely channeled into response planning and adaptation.

#### 5.2.7 Unsustainable and inadequate human resources for RCCE implementation

Human resource constraints limited the scale and consistency of RCCE activities, particularly at the subnational level. While large numbers of CBVs and health workers were deployed in some areas, particularly Lusaka, deployment was often for short periods and was not sustained across all affected locations. Coverage was therefore stronger in some areas than others, with more limited CBV deployment in some affected districts and provinces. Furthermore, limited staffing for the call center resulted in a proportion of calls remaining unanswered. In some provinces, RQA data collectors lacked adequate training in qualitative data collection and note-taking, affecting data quality and subsequent analysis. In addition, delays or non-payment of volunteer incentives in some provinces reduced motivation and engagement.

## 6 Implications for future emergency response planning

The following implications draw on a synthesis of all data sources in the retrospective study and were refined at the time in validation meetings with Collective Service partners, UNICEF ESARO, IFRC, UNZA, UNICEF Zambia (including health and WASH sections), the MoH and ZNPHI. While grounded in the cholera response in Zambia, many of the recommendations have broader relevance and can inform advocacy for integrating RCCE into future emergency responses more generally.

This case study demonstrated several innovations rarely documented in the outbreak response literature:

- The use of RQAs as a first-line analytical tool for operational decision-making.
- Integration of two large-scale community feedback mechanisms into a single multi-pillar dashboard covering all 10 provinces.
- Systematic sharing of RCCE evidence across WASH, case management, logistics and leadership pillars.
- Co-creation of disability-inclusive communication materials in seven languages.
- Cross-pillar adoption of the “3Cs to Stop Cholera” campaign rooted entirely in community-generated insights.

These features make the Zambia response an important global reference for how RCCE can operate as a central outbreak intelligence system.

Drawing on the retrospective study’s analysis of RQA data and community feedback and its use during the cholera response in Zambia, this section presents three interrelated but distinct implications for the governance, institutionalization and multisectoral integration of social and behavioral evidence in future emergency response.

### 6.1 Governance of social and behavioral evidence for RCCE

The Zambia cholera response demonstrates that systematic collection and analysis of social and behavioral data is both feasible and critical to community-centered and contextually appropriate action. Recognition of its value increased over time, particularly as quantitative data alone proved insufficient to guide locally relevant solutions.

Strengthening governance of evidence-based RCCE requires mechanisms to track and document uptake across sectors. Tools such as an Evidence Tracking Framework (ETF) can support systematic monitoring of how RQA and community feedback findings inform decisions, while facilitating retrospective analysis and learning. National academic institutions should be leveraged to generate publishable analyses of impact and lessons learned.

In parallel, multisectoral decision-makers, including those engaged in anticipatory action and climate-related responses, require strengthened capacity to interpret and apply social and behavioral evidence in policy and planning processes, ensuring community perspectives shape early and ongoing response decisions.

### 6.2 Institutionalization of social and behavioral evidence in national systems

Institutionalizing evidence-based RCCE requires sustained investment in evidence systems such as RQAs and CFMs as routine sources of real-time intelligence. This includes strengthening capacity of response actors to collect, analyze, translate findings into operational decisions, and integrating monitoring of behavioral outcomes into response evaluation.

Recent system strengthening efforts include the development of Standard Operating Procedures for incorporating community feedback into routine MoH data systems, and a UNICEF-UNZA-MoH collaboration to develop a practical training course to enhance national and subnational capacity to conduct RQAs. Integrating CFM data into national information systems (e.g., District Health Information Software and the Community Health Information Platform) is critical to streamline information flows, reduce duplication, and embed community voices within routine decision-making processes, beyond individual outbreaks.

Post-response learning should also be institutionalized within health systems, so that lessons from emergencies systematically translated into adapted service pathways, clearer operational protocols, and routine cross-pillar learning mechanisms—rather than treated as one-off reflections. Zambia’s multisectoral cholera response illustrates the value of linking community-response experience with health-system strengthening: reviewing RCCE, WASH, case management, surveillance, logistics, and community feedback systems, and subnational coordination platforms together, after an emergency helps identify what worked, what constrained timely action, and what should be sustained, adapted, or embedded in routine preparedness and response systems (45, 46). This includes reviewing how community concerns influenced changes to care-seeking pathways, ORP and CTC access, transport support, chlorine and soap distribution, safe burial procedures, OCV outreach, and feedback mechanisms—and using these lessons to refine standard operating procedures, referral pathways, training packages, and accountability mechanisms before the next emergency. Institutionalizing this learning would help health systems shift from episodic emergency response to adaptive continuity, where social and behavioral insights gathered during crises routinely inform service delivery, preparedness planning, and multisectoral coordination beyond the immediate outbreak period.

### 6.3 Multisectoral operational integration of RCCE

RCCE must function as a cross-pillar analytical and decision-making resource, rather than a communication add-on. Preparedness planning should ensure RCCE systems and health promotion is maintained *before*, during, and *between* emergencies and pre-positioned financial, human and material resources for RCCE from the outset. Volunteer training, health promotion activities, and community engagement should be maintained outside outbreaks, reducing reliance on *ad hoc* mobilization when emergencies occur. Strengthening community-led engagement and maintaining trusted partnerships across government, civil society and academia further ensures that the collective capacity developed during an emergency is retained for future responses. Engaging local organizations and influencers, such as churches, traditional leaders, district and community committees, and neighborhood clubs enhances trust, relevance, and ownership, and supports the translation of social and behavioral evidence into coordinated multisectoral action.

By establishing regular cross-pillar processes, RCCE can be a critical resource in future emergency responses—helping ensure RCCE data is reviewed to inform decision-making and that evidence gaps are iteratively identified and addressed. A routine communication channel should be established so that emerging community concerns, rumors, access barriers, and service quality issues are rapidly referred to the respective response pillar and tracked to resolution. This can be achieved through joint dashboards, regular IMS updates, and dedicated sessions with response pillars. In the long term, investment in system-building actions—including capacity at national and subnational levels to scale up the existing CFM standard operating procedure with clearly defined accountability—will be crucial.

When integrated across pillars, RCCE intelligence can also strengthen preparedness, anticipatory action and post-emergency response. For preparedness, RCCE can generate real-time information on community alerts, rumors, service access barriers, supply gaps, risk perceptions, misinformation, and priority needs. When systematically collected, analyzed and shared with technical pillars, this intelligence can inform risk analysis, contingency planning, prepositioning of essential commodities, readiness of community feedback systems, and identification of vulnerable or high-risk populations. For anticipatory action, it enables early detection and escalation of community priority needs before conditions deteriorate. In the post-emergency phase, it can support after-action reviews, document lessons learned, track feedback-to-action, address residual misinformation or service barriers, and strengthen systems for future emergencies.

## 7 Transferability and broader relevance of the Zambia experience

The Zambia experience offers valuable lessons for other public health emergency responses, but its transferability should be considered in light of the specific institutional and political context, including support from UNICEF, IFRC, ZNPHI, MoH, UNZA, community-based volunteers, and international technical partners (e.g., UKHSA, US CDC).

Certain elements are likely to be transferable to other settings and emergencies. These include systematically collecting community feedback through rapid qualitative assessments and community feedback mechanisms and other sources, channeling that evidence to relevant technical pillars and decision-makers, and using action tracking frameworks to trace how insights translated into action. Academic institutions can be leveraged to support high-quality rapid qualitative assessments and timely dissemination of community data. The four categories of evidence use that we developed for this study (informing messaging, activities/ capacities, programming, and policy) is an analytic framework that other responses could adopt regardless of their resourcing level.

However, several factors that enabled an evidence-to-action pathway to function effectively in Zambia are less easily replicated. For example, pre-existing partnerships between government (MoH, ZNPHI) and technical/academic institutions (UNZA) that already had working relationships prior to the outbreak, a functioning community feedback dashboard to aggregate and visualize RQA/CFM data in near-real time, technical surge capacity from UNICEF, IFRC, and bilateral partners who leveraged opportunities to facilitate uptake of community insights, and political will and institutional buy-in at the MoH level that enabled rapid uptake of evidence. Settings without comparable institutional and political support structures may find it more difficult to replicate the speed and breadth of evidence uptake observed in Zambia, even with the same commitment to systematically generating community evidence.

## 8 Conceptual and methodological constraints

There were three key limitations to the original retrospective qualitative study. Firstly, while several stakeholders who were consulted mentioned that RCCE evidence had contributed to policy change, concrete examples supported with documentation were difficult to obtain, and therefore the stated examples of how evidence informed policy decisions were not verified; it was not possible to conduct consultations with the MoH at the national level to obtain statements, whether verbal or written, to support these examples. Secondly, the epidemiological data provided by the former ZNPHI National Cholera Coordinator for the temporal analysis did not include geographical disaggregation, and many daily situation reports (SITREPs) were missing so could not be used to build the temporal analysis visual by epidemiological week. Instead, the analysis had to rely on the compiled epidemiological dataset provided. This precluded comparison of cholera cases and case fatality rates between areas with and without RCCE activities. Thirdly, the exact dates (day or epidemiological week) were not available for some RCCE activities and data points. Therefore, elements of the temporal analysis are instead based on the best available information and reasonable approximations. The temporal analysis diagram incorporates contributions from the national interactive CFM dashboard (populated by ZRCS, Lifeline/Childline Zambia (LLCZ), Support to Older People in Zambia (STOP) and Zambia Interfaith Networking Group Organization (ZINGO)), but the diagram excludes CFMs operated by other partners during the response. It is also important to note that the CFM dashboard is no longer operational, which limited the availability of dates for RCCE activities in the temporal analysis visual, particularly for CFM data.

## 9 Conclusion

This case study demonstrates that social and behavioral evidence can play an important role in outbreak response when it is generated rapidly, integrated into decision-making, and acted upon across response pillars. In Zambia’s cholera response, this evidence informed communication and messaging, community engagement, adaptation of RCCE activities, multisectoral programming, and even policy decisions that improved the relevance and feasibility of interventions. The findings reinforce the need to position evidence-based RCCE as a core response function and to strengthen the systems, coordination, and capacities needed to translate community-generated insights into action in future public health emergencies.

## Data Availability

The original contributions presented in the study are included in the article/Supplementary material, further inquiries can be directed to the corresponding author.

## Footnotes

1 Response pillars have been renamed and restructured at different times. The Zambia Multisectoral Cholera Elimination Plan: 2019–2025 lists only 6 pillars (10) while the Zambia Multisectoral Cholera Outbreak 2023/2024 Response Intra-Action Review Report names (10) pillars (18).
