# Supplementary material for: From listening to action: how social and behavioral evidence informed multisectoral cholera response in Zambia, 2023–2024
Source: Front Public Health. 2026 Oct 9;14:1895133. doi: 10.3389/fpubh.2026.1895133 (PMC13690732; doi:10.3389/fpubh.2026.1895133)
Supplement: Supplementary file 1 [file Table_1.DOCX]

Supplementary file 1: Temporal analysis of cholera cases and key RCCE activities during the 2-23-24 cholera outbreak in Zambia.
