# Supplementary material for: From listening to action: how social and behavioral evidence informed multisectoral cholera response in Zambia, 2023–2024
Source: Front Public Health. 2026 Oct 9;14:1895133. doi: 10.3389/fpubh.2026.1895133 (PMC13690732; doi:10.3389/fpubh.2026.1895133)
Supplement: Supplementary file 2 [file Table_2.DOCX]

Supplementary file 2: Thematic analysis of the use of evidence to inform Risk Communication and Community Engagement (RCCE) actions

| **Feedback type/platform** | **Themes** | **Insight** | **Action taken** |
| --- | --- | --- | --- |
| *RCCE evidence informed risk communication and messaging (n = 16)* | | | |
| ZNPHI call center | Introduction of new messages | Many people requested the same information about cholera | ZNPHI provided answers to common questions to be addressed by call center agents.  ^53^ |
| Peer-to-peer health promotion among older persons, targeted outreach to PLWDs and refugee communities in Lusaka | Introduction of new messages | There were misconceptions about foreigners causing cholera | STOP, in collaboration with the MoH and UNICEF, supported volunteers in tailoring messages to these insights.  ^83^ |
| RQAs | Introduction of new messages  Cross-pillar work  Updating of existing materials (e.g., CBV job aids, FAQs) | The community was reluctant to seek care at CTCs due to fears of dying. People’s reasons for fearing CTCs varied by location, but were in some cases fueled by fear campaigns on social media | Targeted education and messaging campaigns were developed through cross-pillar work with Case Management to build trust in CTCs and encourage early care-seeking. CBV job aids were developed including information on what happens at CTCs and ORPs.  ^5,15,32,36,53,60^ |
| RQAs | Introduction of new messages  Updating of existing materials (e.g., CBV job aids, FAQs)  Creation of targeted IEC materials for specific groups | There were misconceptions within the community about cholera transmission and the use of traditional remedies/prevention methods such as kachasu (local home brew), the krau krau method of using Vaseline on a cloth as protection, leaves and prayer | The RCCE pillar tailored their health communication messages and created IEC materials, including clarifying transmission routes and promoting proven prevention and treatment methods like ORS. This included updating the CBVs’ Q&A tool and training materials and distributing child-friendly posters in schools about the use of kachasu.  ^5,32,50–55^ |
| RQAs | Introduction of new messages  Updating of existing materials (e.g., CBV job aids, FAQs)  Uptake and dissemination by the MoH | Communities had mixed awareness of ORS and how to properly prepare it and could not afford it | Clear messaging was created around the use of ORS as the first line of treatment before seeking health care. This was communicated as part of the 3Cs messaging and CBV job aids and training. Information about ORS was shared on social media by the MoH and on television.  ^32^ |
| RQAs | Introduction of new messages  Updating of existing materials (e.g., CBV job aids, FAQs) | Communities did not know how to find ORPs or the services they provided. CBVs did not have adequate knowledge about ORPs | UNICEF created ORP job aids to help people locate services. These were given to CBVs to share with community members.  ^15,36,52,56^ |
| RQAs | Introduction of new messages  Updating of existing materials (e.g., CBV job aids, FAQs) | Although there was high acceptance of the OCV in principle, most people were unaware of the vaccine. Communities thought the vaccine was for polio and there was also low awareness of how long OCV was effective and where it was available | IEC materials were developed about OCV. UNICEF developed OCV job aids for CBVs and HWs to raise awareness about the vaccine. These materials continued to be used during later outbreaks in Dec 2024 and April 2025.  ^15,32,36,56^ |
| Door-to-door visits by CBVs, EHS, CHWs and doctors | Refocused health messaging | People were confused about when to present to the health facility because the focus of messaging was on the last symptom rather than the first | The health message was changed to focus on the first symptom of cholera, in a culturally acceptable way.  ^60^ |
| RQAs | Refocused health messaging  Uptake and dissemination by the MoH | Communities had some correct knowledge about cholera and had heard a range of cholera prevention guidance, but often the greatest emphasis was on a clean environment and there were knowledge gaps | The Zambia-specific ‘3Cs’ information campaign was jointly developed by UNICEF, MoH and ZNPHI, focusing on clean water, clean hands and early care-seeking. A poster was printed and distributed widely in Lusaka and other provinces. The shift to focus on 3Cs has been emphasized in MoH statements about the outbreak.  ^5,7,14,15,32,36,53,56,58,63,84^ |
| CFM dashboard/RQAs | Refocused health messaging  Updating of existing materials (e.g., CBV job aids, FAQs)  Uptake and dissemination by the MoH | The CFM dashboard facilitated gap identification for WASH actors by helping them identify which messages were ‘landing’ | WASH actors adjusted the outreach activities they provided alongside distribution of hygiene kits. Hygiene promotion messages were intensified, and communities were sensitized on how to use chlorine correctly. The messages were integrated into CBV door-to-door and routine PA sensitizations, and relevant job aids developed for CBVs. The MoH also shared social media posts on how to use chlorine.  ^5,32,56^ |
| RQAs | Creation of targeted IEC materials for specific groups  Multichannel dissemination of messages | IEC materials were inaccessible for people with disabilities (e.g., deaf and blind) | A co-creation workshop was held with disability networks and organizations of PLWDs, including the Zambia Deaf Society and STOP, resulting in finalized RCCE materials: OCV poster, ORP and OCV job aids, cholera brochures, and translated 3Cs posters (braille and sign language), all distributed in hotspot areas. Revised multimedia messages were disseminated via 15 national and community radio/television stations with over 10 million estimated listeners. STOP introduced a sign language interpreter for television dissemination.  ^15,32,36,56,83^ |
| RQAs | Creation of targeted IEC materials for specific groups | Communities were concerned about cholera risks as children returned to school. | UNICEF developed child-friendly IEC materials.  ^32^ |
| Peer-to-peer health promotion among older persons, targeted outreach to PLWDs and refugee communities in Lusaka | Creation of targeted IEC materials for specific groups | IEC materials were not available in appropriate languages | RCCE materials were translated into Iswatini, Swahili and other languages, and disseminated.  ^83^ |
| RQAs | Multichannel dissemination of messages | Community members highlighted the transportation barriers they faced in reaching CTCs, including lack of transportation, impassable roads and safety concerns | To accompany transport solutions, communities were sensitized via different platforms (radio, television, PA and door-to-door) on how to get urgent transfers to CTCs.  ^32^ |
| RQAs and other community feedback | Creation of IEC materials and job aids | People provided feedback, concerns and questions about cholera via various feedback channels | Q&A document developed, intended to be updated regularly and kept as an open-source document of Q&As addressing the main feedback and data insights trends. The document is to be accessed and used both by decision-makers and community-based implementers (e.g., CBVs).  ^58,85^ |
| Lifeline/Childline call center | Updating of existing materials (e.g., CBV job aids, FAQs) | People reported fears about cholera, issues of access to services | Call center feedback helped Lifeline/Childline adapt the FAQs used by call center agents.  ^86,87^ |
| *RCCE evidence informed the development adaptation or strengthening of RCCE activities/capacities (n = 8)* | | | |
| Door-to-door visits by CBVs  RQAs | Targeted engagement of specific groups  Adaptation of response plans and budgets | Although a high-risk group, it was noted that men were not involved in CBV door-to-door visits as they are not home during the day | The RCCE response plan and budget were adjusted to include strategies to reach men with risk information. Subsequent rounds of RQAs were conducted by UNICEF with men in affected communities, and the insights were used to inform programming, including targeted interventions for men conducted in hotspots together with the DL&R pillar. ZRCS and partners (Global Health Corps, Keepers Foundation, etc.) conducted sensitizations and distributed IEC materials in public spaces.  ^32,58^ |
| RQAs | Targeted engagement of specific groups  Uptake and dissemination of messaging by the MoH | It was found that communities were using kachasu (local home brew) as a first-line prevention and treatment for cholera | Young people and men were engaged via community dialogue meetings and sensitizations to address this. The Minister of Health visited cholera hotspots and sensitized communities against the use of kachasu to treat cholera.  ^32^ |
| RQAs | Targeted engagement of specific groups | Although there was high acceptance of the OCV in principle, most people were unaware of the vaccine, and particularly men | Men and young people were engaged with OCV sensitization via targeted activities (e.g., in bars, bus stations and schools) and older people were engaged through STOP Zambia.  ^32,34^ |
| RQAs | Targeted engagement of specific groups | Specific needs, challenges and barriers existed for certain groups, such as fishermen | Community dialogue meetings were held with at-risk communities to understand trusted channels of communication as well as preferred times for engagement.  ^32^ |
| RQAs | Partnership with religious and community leaders | It was identified that churches played a big role in disseminating information and that they did not have IEC materials | WHO engaged the churches to provide RCCE announcements and encouraged them to create posters for cholera based on previous examples from COVID-19.  ^60^ |
| RQAs | Partnership with religious and community leaders | Some communities reported stigmatizing attitudes towards families affected by cholera | ZINGO supported religious leader involvement across hotspot churches in Lusaka to address stigma. ZRCS supported anti-stigma campaigns with local drama groups. With the support of ZNPHI, ZRCS also integrated mental health and psychosocial support activities in the cholera response.  ^32^ |
| CFM | Strengthening RCCE coordination and capacity | Localized gaps in RCCE capacity were identified | The MoH asked response partners to strengthen RCCE capacities of provinces and districts with national borders. Luapula province was prioritized, and five districts were trained on RCCE, RCCE pillar activation and coordination, CFMs, and RCCE reporting in emergencies.  ^7^ |
| RQAs | Adaptation of response plans and budgets | The RCCE response plan was focused on figures and budget but lacked social and behavioral insights | The RCCE response plan was updated to include diverse strategies and approaches based on insights from the RQAs.  ^52,58^ |
| *RCCE informed programming/operational decisions of other pillars (n = 13)* | | | |
| RQAs | Improving access to health services  Uptake of RCCE evidence across pillars  Uptake of RCCE evidence by government  Capacity building for front-line staff | Community members highlighted the transportation barriers they faced in reaching CTCs, including lack of transportation, impassable roads and safety concerns. Initially the only CTC was at Heroes’ Stadium in Lusaka, and people were afraid to go there | A transport funding scheme was developed and transportation provided to help facilitate access to healthcare. The MoH and UNICEF paid to rehabilitate 7 ambulances in Lusaka for patient transport and provided fuel. Other partners purchased additional vehicles, working with private car hires. Transportation was also provided to patients upon discharge. MoH also created more CTCs and ORPs to make services more accessible for people in communities and to reduce fear in accessing these. CBVs were able to refer suspected cases to health facilities.  ^5,7,32,34,36,50,56,58,60,80,84^ |
| RQAs | Addressing socio-economic barriers to care  Uptake of RCCE evidence across pillars  Uptake of RCCE evidence by government | Communities had concerns regarding the costs of cholera prevention (including chlorine, soap, etc.) and treatment-seeking, as well as burials. Families in the poorest communities were not coping with the economic burden of cholera | This evidence supported broader discussions with other government ministries to activate emergency support mechanisms. UNICEF and the Zambian Social Welfare System established an emergency cash transfer program (Public Welfare Assistance Scheme) to provide 900-1200 kw to 400 households throughout Lusaka to address cost concerns raised in the RQAs. ADRA established a cash transfer system that will reach 2,000 households.  ^5,32,36,58^ |
| RQAs | Addressing socio-economic barriers to care  Uptake of RCCE evidence across pillars  Improving WASH | People were unable to purchase soap and chlorine | UNICEF immediately procured chlorine and soap as part of Stop Cholera kits and provided the chlorine and soap to Red Cross Volunteers for distribution. The WASH team distributed chlorine packages. Other partners such as ADRA also distributed hygiene kits, including chlorine bottles.  ^15,32,36,51,52,54,56,58,74,84^ |
| RQAs | Strengthening feedback mechanisms  Uptake of RCCE evidence across pillars  Uptake of RCCE evidence by government | Relatives of patients at Heroes Stadium CTC had no contact with family members and there was no identification information for patients. This contributed to reluctance to present at the CTCs | The MoH and ZNPHI developed a feedback mechanism for patients’ relatives at CTCs, introduced daily patient updates for families and created a patient registry. A phone system was set up to call relatives to provide feedback.  This also facilitated identification of the deceased.  ^15,36,50,56,60^ |
| RQAs | Improving PSS  Uptake of RCCE evidence across pillars | Communities were opposed to the barriers to engaging in traditional burial rituals and could not grieve appropriately. Burials were costly and traumatic, and there was little or no psychosocial support for grieving families | Based on this finding, family was engaged in the burial process and selected family members allowed to participate. In addition, psychosocial support was provided to bereaved families by ZRCS through volunteers.  ^32,36,60,62,63,88^ |
| RQAs | Uptake of RCCE evidence across pillars  Improving WASH | Communities were concerned about cholera risks as children returned to school | WASH/Education completed assessments and UNICEF provided WASH supplies to priority schools.  ^32^ |
| RQAs | Improving access to health services  Uptake of RCCE evidence across pillars | There were high levels of willingness to take OCV, but it was only delivered at static sites during working hours. This was of particular concern for men, who could not access sites during working hours | Distribution of OCV was expanded to include households, churches, community spaces and after hours. Findings from the RQA were shared with WHO two days after completion of the first round of RQAs, and the distribution plan was adapted immediately to be reflected in the OCV campaign that began that same day.  ^32,34,59^ |
| RQAs | Addressing socio-economic barriers to care  Uptake of RCCE evidence across pillars | Communities had mixed awareness of ORS and how to properly prepare it and could not afford it | CTCs provided discharge kits, CBVs distributed ORS household cholera kits to communities via household distributions and ORCs/ORPs.  ^32^ |
| RQAs | Improving WASH  Uptake of RCCE evidence across pillars  Capacity building for front-line staff | Poor hand hygiene, overuse of chlorine, and other poor IPC practices were observed in CTU/CTCs. There were also insufficient human resources in the CTU/CTCs | IPC training was conducted for HWs in Lusaka.  ^32^ |
| CFM dashboard | Strengthening feedback mechanisms  Uptake of RCCE evidence by government  Capacity building for front-line staff | The value of community feedback was recognized by the MoH for informing Incident Management Team decisions and reviewing community programs | MoH Health Promotion officers (HPO) were engaged to prioritize use of the community feedback data at the health facility level to inform Incident Management Team decisions, reviewing their community programs and addressing any issues raised by communities through the CFM in a timely manner.  ^7^ |
| CFM dashboard/RQAs | Improving WASH  Uptake of RCCE evidence across pillars | WASH infrastructure and services were lacking (e.g., toilets, drains, bores, rubbish collection) | WASH recommendations such as garbage collection were provided and adopted. ZRCS used localized feedback to target interventions for construction of waterborne toilets, drilling of bores and upgrading water points to a solar powered system, including in health facilities. UNICEF supported water companies to repair their water provision systems.^52^ |
| Lifeline/Childline call center | Immediate action for individual cases | Families became separated due to guardians contracting cholera, leaving children without care | These reports were escalated to the appropriate agencies for immediate action.  ^5,32,52,56^ |
| Lifeline/Childline call center | Capacity building for frontline staff  Improving PSS  Immediate action for individual cases | A gap was identified in the provision of psychosocial support to people affected by cholera or health staff at CTCs.  People faced stigma in the community on being discharged | Counsellors were placed at the Heroes Help Desk and communities were made aware of the Lifeline/Childline toll-free lines. Call center agents were trained on cholera information, while ZNPHI through ZRCS integrated MHPSS into the cholera response. Lifeline/Childline followed up on individual cases needing psychosocial support. They also introduced CBVs to their programming to do community-based care and follow up.  ^32,86^ |
| *RCCE evidence informed policy decisions (n = 2)* | | | |
| RQAs | Uptake of RCCE evidence by government  Adapting preventive measures to accommodate social and cultural considerations | People were dissatisfied with being excluded from burial rituals, with many families feeling they could not grieve appropriately. The MoH faced challenges in identifying deceased bodies. | According to the Interagency RCCE Action Tracker, the national safe and dignified burial guidelines were revised by the MoH to allow a select number of family members to participate in burial rituals and this change was communicated to communities. We do not have written evidence of this policy change. This change also aimed at enabling families to help identify deceased bodies, as identification had been challenging without their presence. The establishment of an information/help desk at Heroes Stadium also assisted with identification.  ^8,32,34,36,60–63^ |
| CFM | Uptake of RCCE evidence by government  Improving WASH | Communities were using pit latrines and shallow wells, which were contributing to the spread of cholera | A policy directive was issued to mandate the emptying of pit latrines and burying shallow wells in cholera hotspot areas, supported by civil society contributions of chlorine and fuel to expedite the exercise, while the government continued to provide water tanks as an alternative safe water source.  ^64–66^ |
